# Hooper’s Physician’s Vade Mecum

**Published:** 1884-09

**Authors:** 


HOOPER'S PHYSICIAN'S VADE MECUM, A Manual of the Principles and Practice of Physic, With an Outline of General Pathology, Therapeutics and Hygiene; tenth edition; in two volumes. Revised by William Augustus Guy, M. B., Cantab. F. R. S., and John Harley, M. D., London, F. L. S. William Wood & Co., New York, 1884.

Like old wine, this book comes to us recommended by its great age, having been published first in 1823, and doubtless this accounts for its containing a great amount of crude material which is not abreast of the progress of the present day. Notwithstanding the modifications and additions by the revisers, it would be more appropriately styled a hand-book for students than a companion for the practitioner.

It is notable that in the classification of remedies, under the heading of inhalations, no allusion is made to volatile alkali, sulphuric ether, chloroform, nitrite of amyl, bromide of ethyl, etc., which are used in many cases independent of any general anesthetic effects. The formulæ are numerous and varied so as to be useful to the student rather than to the physician.

Should some of the utilitarian practical spirit of American authorship be employed in reducing these two volumes to one by expunging all that does not pertain to a true *vade mecum* of the physician, they would not suffer any detriment, and be far more convenient for reference.

